# Revealing the effect of Nb or V doping on anode performance in Na_2_Ti_3_O_7_ for sodium-ion batteries: a first-principles study†

**DOI:** 10.1039/d3ra01755a

**Published:** 2023-06-05

**Authors:** Suk-Gyong Hwang, Chung-Hyok Kim, Song-Hyok Choe, Kum-Chol Ri, Chol-Jun Yu

**Affiliations:** a Computational Materials Design, Faculty of Materials Science, Kim Il Sung University PO Box 76 Pyongyang Democratic People’s Republic of Korea cj.yu@ryongnamsan.edu.kp

## Abstract

Sodium titanate Na_2_Ti_3_O_7_ (NTO) has superior electrochemical properties as an anode material in sodium-ion batteries (SIBs), and Nb or V doping is suggested to enhance the electrode performance. In this work, we carry out systematic first-principles calculations of the structural, electronic and electrochemical properties of NTO and Na_2_Ti_2.75_M_0.25_O_7_ (M = Nb, V), using supercells to reveal the effect of Nb or V NTO-doping on its anode performance. It is found that Nb doping gives rise to the expansion of cell volume but V doping induces the shrinkage of cell volume due to the larger and smaller ionic radius of the Nb and V ions, respectively, compared to that of the Ti ion. We perform structural optimization of the intermediate phases of Na_2+*x*_M_3_O_7_ with increasing Na content *x* from 0 to 2, revealing that the overall relative volume expansion rate is slightly increased by Nb and V doping but remains lower than 3%. Our calculations demonstrate that the electrode potential of NTO is slightly raised and the specific capacity is reduced, but the electronic and ionic conductivities are improved by Nb or V doping. With the revealed understanding and mechanisms, our work will contribute to the search for advanced electrode materials for SIBs.

## Abstract 2

First-principles simulations of sodium titanate Na_2_Ti_3_O_7_ with Nb- or V-doping, have been performed to identify the variation of structural, electronic and electrochemical properties for use in sodium-ion batteries.

## Graphical abstract

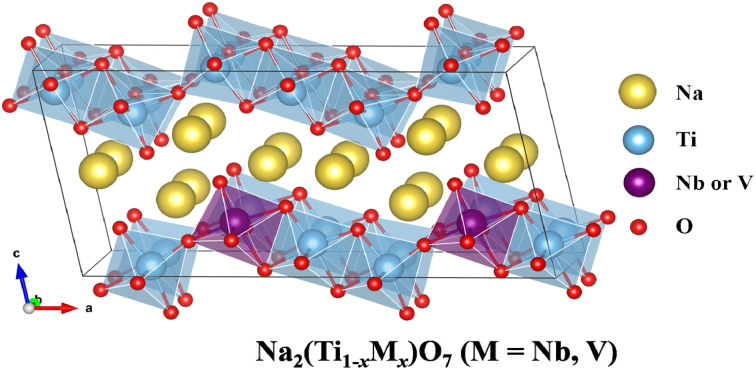

## 1 Introduction

Recently, increasing attention has been paid to developing and utilizing renewable and clean energy sources such as solar energy, wind power and tidal power, to mitigate the serious challenges of global warming and the energy crisis. For progress, an indispensable prerequisite is to develop large-scale electrochemical energy storage (EES) systems that are low cost, high performance and have a long lifetime.^1^ Among the various EES devices, lithium-ion batteries (LIBs) are highly promising due to their high energy density and long-term cycling life, and are widely used for portable electronic appliances and electric vehicles. However, the limited reserves of Li resources have caused a rapid increase in cost, making them unsuitable for large-scale EES systems.^2,3^ In this context, there is renewed interest in sodium-ion batteries (SIBs) as a promising alternative to LIBs due to the global abundance and low cost of Na resources.^4–6^ To realize commercially viable SIBs, however, suitable electrode materials need be developed.^7,8^ Although graphite is a typical intercalation-type anode material for LIBs, it is not suitable for SIBs because of the larger radius of the Na ion (1.02 Å) compared to the Li ion (0.76 Å), which has created an intensive search for alternative co-intercalation^9–11^ and hard carbon materials.^12–14^

Sodium titanates with the general chemical formula *n*Na_2_O–*m*TiO_2_, characterized by layered or tunnel structures, have been adopted as promising insertion-type anode materials for SIBs due to their low cost, low electrode potential and non-toxicity.^15–20^ Nanophase titanium dioxide, TiO_2_ (*n* = 0), with rutile,^21^ anatase,^22^ hollandite^23^ and trigonal bipyramid^16,24^ structures has shown reversible capacities of 100–150 mA h g^−1^ due to the Ti^3+/4+^ redox couple. However, these compounds have a relatively high electrode potential of over 0.5 V and lower rate capability compared to hard carbon. The tunnel-structured sodium titanates such as NaTi_2_O_4_,^25^ Na_2_Ti_4_O_9_ (ref. 26) and Na_2_Ti_6_O_13_ (ref. 27–30), have also been reported to show reversible sodium insertion/extraction capacities of 45 and 66 mA h g^−1^ at a high current rate of 2 A g^−1^ (30C) with long cycling life (5000 cycles). Such high-rate capability and good cyclability can be associated with the low barrier for Na ion diffusion (0.24 ∼ 0.44 eV) due to the existence of large tunnels.^28^ However, the higher electrode potential of 0.8 V *vs.* Na/Na^+^ counter electrode has been found to be a major limiting point for anode applications of SIBs.

A remarkably low electrode potential of 0.3 V has been observed in step-layered sodium titanate Na_2_Ti_3_O_7_ (NTO), together with a high capacity of 178 mA h g^−1^ (theoretically 311 mA h g^−1^),^31–33^ being comparable with hard carbon. The crystalline structure of NTO is characterized by step layers of edge-sharing TiO_6_ octahedra, which can accommodate 2 Na ions per formula unit, transforming into Na_4_Ti_3_O_7_ with a minor lattice expansion of ∼6%. In spite of the intrinsic benefit of its layered structure to ion diffusion, the rate capability of batteries is limited by the sluggish kinetics of Na ions and poor electronic conductivity.^33,34^ Three strategies have been developed to address this issue. Similar to other electrode materials, the first method is to use nanosize NTO made through elaborate synthesis routes, this shortens the transport path for ions and electrons.^35–40^ However, a plateau voltage profile was often lost, and instead, a slope-like voltage profile was observed, indicating pseudocapacitance anode behaviour for sodium-ion capacitors (SICs).^41^ The second method is to introduce conductive layers such as carbon^42,43^ and its derivatives^44,45^ to enhance electronic conductivity. Doping or substituting Ti in NTO has been proved to be an effective third method for facilitating ionic and electronic transport.^46–50^ In particular, Nb doping into NTO has shown an increase in unit cell volume and lower band gap, resulting in enhancement of rate capability to 89 mA h g^−1^ at 5C and cyclability to 78.5%, after 500 cycles.^48^ Through first-principles calculations using density functional theory (DFT) framework, it was revealed that Ti/V exchange in NTO led to a lowered band gap and electrode potential with increased capacity.^50^ However, no comprehensive study of Nb- or V-doped NTO has yet been reported, but there are a few theoretical studies for NTO.^15,32–34,50^

In this work, we perform systematic first-principles calculations on structural, electronic and electrochemical properties of Nb- and V-doped NTO compounds towards advanced anode materials for SIBs. To simulate the doping, we construct supercells for NTO – Nb-doped NTO (NTNO) and V-doped NTO (NTVO) – and apply the first-principles pseudopotential plane wave method as described in Section 2. The calculation results for the structural properties are provided in Subsection 3.1, and the energetic and electrode properties are given in Subsection 3.2. Subsections 3.3 and 3.4 explain the results for electronic and ionic conductivities, respectively. Finally, main conclusions are provided in Section 4.

## 2 Materials modeling and computational methods

### 2.1 Modeling

**Fig. 1:**
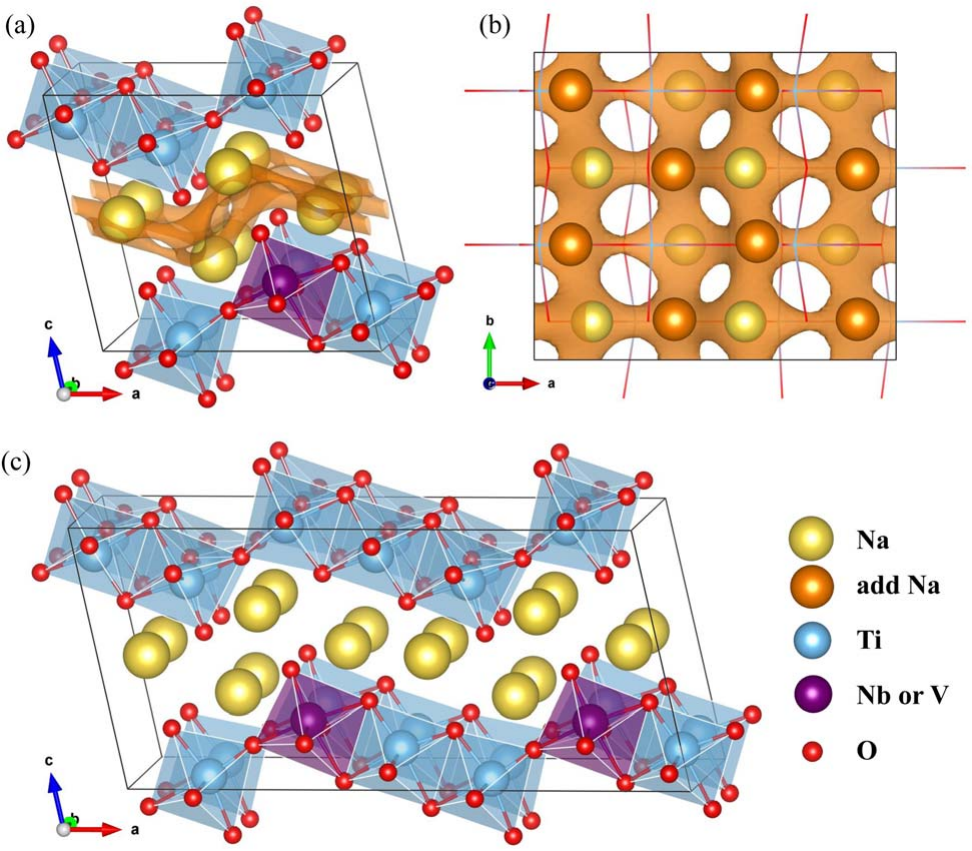
Crystalline structures of Nb- or V-doped Na_2_Ti_3_O_7_. (a) Perspective view of supercell built by doubling the unit cell in the *b* direction, and (b) its top view. Brown-colored isosurface map of bond valence sum difference (ΔBVS) with a value of 3, is shown to indicate the positions of the added Na ions and their diffusion path. (c) Perspective view of a 2 × 2 × 1 supercell for simulating Na ion diffusion.

The NTO crystal are a monoclinic system with space group *P*2_1_/*m*.^51^ The unit cell with lattice constants of *a* = 9.118 Å, *b* = 3.795 Å, *c* = 8.563 Å, *α* = *γ* = 90° and *β* = 101.55° (ref. 48), contains 2 formula units (24 atoms). Titanium and oxygen ions form edge-sharing TiO_6_ octahedra making a step layer – the step layers are connected with each other by a sharing vertex – and sodium ions are placed in the interlayer space with two different coordinations of 7 and 9. To investigate the doped structures and Na insertion/desertion, we built a supercell by doubling the unit cell in the crystallographic *b* direction (4 formula units, 48 atoms), as depicted in Fig. 1(a) and (b). The migration of Na ions was simulated using 2 × 2 × 1 supercells (8 formula units, 96 atoms) shown in Fig. 1(c).

The doped structures can be realized by replacing Ti with transition metal (TM) M in the Na_2_Ti_3_O_7_ crystal, where M represents one or more 4d and 3d TMs. Among the possible elements such as Nb, Mn (4d TMs) and V, Cr, Mo (3d TMs), Nb and V are preferred in this work. In the NTO unit cell, there are three different Ti positions. Therefore to fix the Ti/M (M = Nb, V) exchange position, we constructed the three different supercells where one of the 12 Ti ions was replaced by Nb or V, and performed structural optimization to select the one with the lowest total energy.^50,52^ The resultant compounds have the chemical formula Na_2_Ti,_2.75_M,_0.25_O_7_.

In Na_2_Ti_3_O_7_, all the Ti ions have an oxidation state of +4 and some of them can be reduced to the +3 oxidation state upon insertion of Na ions for intercalation-type electrode application of SIBs. The preliminary positions of inserting Na ions can be identified by plotting the isosurface map of the bond valence sum difference (ΔBVS), calculated by 
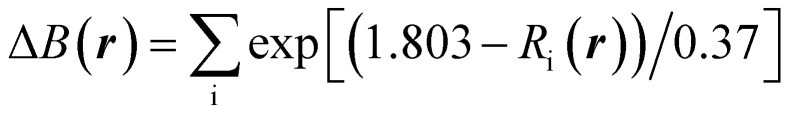
 where *R*_i_(***r***) = |***r*** − ***R***_i_|, where ***R***_i_ is the position vector of the i-th atom for the Na–O bond.^53,54^ As shown in Fig. 1(a) and (b), there are 8 additional positions for Na ions in the doubled supercell, and we expect the final product Na_4_Ti_3_O_7_ with a specific capacity of 178 mA h g^−1^ in accordance with experimental work.^31^ Eventually, one third of the total Ti ions retain the +4 state (Ti^4+^2Ti^3+^). We note that Na_5_Ti_3_O_7_ with all Ti^3+^ ions (311 mA h g^−1^) is not practically allowed due to the severe deviation of the cell.^32,50^ For the Nb- and V-doped NTOs, the same process is suggested with transformation M^4+^ → M^3+^, based on experimental findings of a similar specific capacity to NTO.^48^ Considering that Nb and V have a +5 oxidation state as well, they might be in either M^5+^ or M^4+^ ionic states after doping into NTO. For the former circumstance, one Ti^4+^ ion could be transformed into a Ti^3+^ ion for charge compensation, which nevertheless would not affect the Na insertion process suggested above.

### 2.2 Computational details

All the DFT calculations were performed using the pseudopotential plan wave method as implemented in the Quantum ESPRESSO (version 6.5) package.^55^ The ultrasoft pseudopotentials provided in the GBRV library^56^ were used to describe the electrostatic interaction between the ionic core and valence electrons with valence electron configurations of Na-2s^2^2p^6^3s^1^, Ti-3s^2^3p^6^3d^2^4s^2^, Nb-4s^2^4p^6^4d^4^5s^1^, V-3s^2^3p^6^3d^3^4s^2^ and O-2s^2^2p^4^. The Perdew–Burke–Ernzerhof for solids (PBEsol) formulation within the generalized gradient approximation was adopted for the exchange–correlation (XC) interaction.^57^ To take account of the localized 4d or 3d electrons of the TM elements, we used the DFT + *U* method,^58^ with the effective Hubbard parameter *U*_eff_ = 3.0 eV for Ti, Nb and V, consistent with previous DFT work.^15,50,59,60^ As the main computational parameters, the kinetic cutoff energies were set to 60 and 600 Ry for the wave function and electron density, respectively, and the *k*-point meshes for the Brillouin zone (BZ) integration were set to (2 × 2 × 2) for structural optimizations and (4 × 4 × 4) for density of states (DOS) calculations. The structural optimizations were finished when the atomic forces and the lattice pressure converged to 5 × 10^−4^ Ry bohr^−1^ and 0.05 kBar, respectively. These computational parameters guarantee the total energy accuracy of 5 meV per atom. Spin-polarization was considered in the DOS calculations.

To simulate the Na ion diffusion, we used the 2 × 2 × 1 supercells, which allows Na ion migration along the *a* and *b* directions, and only the Γ point for the BZ integration. The activation energies for the vacancy-mediated Na ion diffusions and the corresponding diffusion paths were determined by applying the climbing image nudged elastic band (NEB) method.^61^ The number of NEB image points was set to 7 along the migration path. During the NEB runs, the lattice constants of supercells were fixed, while all the atoms were relaxed until the forces converged to 0.01 eV Å^−1^. The crystal structures were visualized using the VESTA code.^62^

## 3 Results and discussion

### 3.1 Structural properties

Firstly, the lattice parameters of the NTO unit cell with 2 formula units (24 atoms) were determined by performing structural optimization using the PBEsol functional. The optimized lattice constants are *a* = 9.089 Å, *b* = 3.800 Å, *c* = 8.532 Å and *β* = 101.82°, which are in good agreement with the experimental values of *a* = 9.118 Å, *b* = 3.795 Å, *c* = 8.563 Å and *β* = 101.55°.^48^ The relative errors are less than ±0.6%, indicating that our selections for computation, including the pseudopotentials, XC functional and *U*_eff_ values, can provide reliable results for electrochemical and electronic properties.

With the same computational settings, the structural optimization of the doubled supercells with 4 formula units (48 atoms) for NTO, NTNO and NTVO were then performed. Table 1 lists the optimized lattice constants and cell volumes in comparison with the available experimental data. As mentioned above, the configuration with the lowest total energy was selected among the three different configurations for the doped NTO supercells. In Table 1, we list the experimental lattice constants for Na_2_Ti,_2.97_Nb,_0.03_O_7_,^48^ which has a significantly lower concentration of doping Nb ions compared with our case of Na_2_Ti,_2.75_Nb,_0.25_O_7_. It was found that the cell volume was slightly expanded by Nb doping with a relative volume expansion rate of 0.91%, estimated by *r*_vol_ = (*V*_NTNO_ − *V*_NTO_)/*V*_NTO_ × 100%, in accordance with the experiment. This value of *r*_vol_ is larger than the value of 0.19% estimated from experiments, due to the higher concentration of doping Nb ions in this work. The volume expansion by Nb doping is attributed to larger ionic radius of Nb^4+^ (0.68 Å) or Nb^5+^ (0.64 Å) and Ti^3+^ (0.67 Å) than that of Ti^4+^ (0.605 Å). In contrast, V doping was found to shrink the cell volume with a *r*_vol_ value of 0.42% because of smaller ionic radii of the V^4+^ (0.58 Å) and V^5+^ (0.54 Å) ions.

**Table 1:** Optimized lattice constants and volume of doubled supercells (4 formula units, 48 atoms) of compounds

Compounds	*a* (Å)	*b* (Å)	*c* (Å)	*β* (deg.)	Volume (Å^3^)
Na_2_Ti_3_O_7_	9.090	7.610	8.513	101.99	576.018
Na_2_Ti_3_O_7_ a	9.118	7.590	8.563	101.59	580.608
Na_2_Ti,_2.75_Nb,_0.25_O_7_	9.115	7.610	8.555	101.60	581.259
Na_2_Ti,_2.97_Nb,_0.03_O_7_ a	9.121	7.593	8.575	101.59	581.728
Na_2_Ti,_2.75_V,_0.25_O_7_	9.068	7.581	8.531	102.01	573.605

a Taken from ref. 48.

**Fig. 2:**
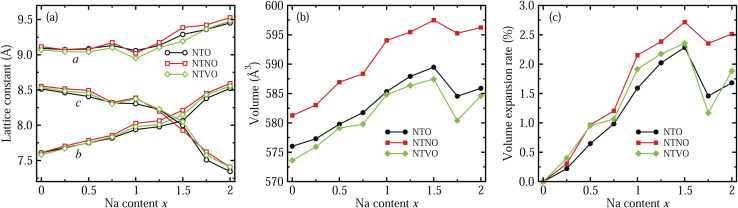
Variation tendency of (a) lattice constants (*a*, *b* and *c*), (b) supercell volume, and (c) volume expansion rate in the doubled supercells for Na_2+*x*_Ti_3_O_7_ (NTO), Na_2+*x*_Ti,_2.75_Nb,_0.25_O_7_ (NTNO) and Na_2+*x*_Ti,_2.75_V,_0.25_O_7_ (NTVO), with increasing Na content *x* from 0 to 2.

In the next step, we identified the intermediate phases formed by inserting additional Na ions into the host compounds NTO, NTNO and NTVO, for suitability in the charge/discharge process in SIBs. As expected from ΔBVS analysis, the content of additional Na ions could be allowed to vary between 0 and 2, forming intermediate phases of Na_2+*x*_TO, with insignificant structural variation. Fig. 2 shows the variation tendencies of lattice constants and volumes of the doubled supercells for Na_2+*x*_TO, Na_2+*x*_TNO and Na_2+*x*_TVO, with increasing Na content *x* from 0 to 2 with an interval of 0.25. It should be noted that there exists numerous configurations for Na insertion at each value of Na content *x*, and the lowest energy configuration was picked out with performing structural optimization (see Fig. S1–S3 for final configurations).†

For all three compounds, the lattice constants *a* and *b* were found to increase gradually with increasing Na content *x*, whereas the lattice constant *c*, *i.e.*, the interlayer distance, was found to decrease gradually until *x* = 1.5 and then it decreased rapidly, as shown in Fig. 2(a). Of course, some wavy features were observed. This indicates that the interaction between the step layers can be enhanced *via* the attractive Na–O ionic bonding by insertion of additional Na ions into the interlayer space, while the repulsion between Na ions and TM ions is dominant in the lateral directions. As can be seen in Fig. 2(b), the supercell volumes were found to overall increase with increasing Na content *x* from 0 to 1.5, then the volumes decrease abruptly at *x* = 1.75 and again increase at *x* = 2 for NTO, NTNO and NTVO. The abrupt decrease of volume at *x* = 1.75 is associated with the rapid decrease of the interlayer distance due to the enhancement of layer–layer interactions. Across the whole range of Na content, the volumes of Nb-doped NTO supercells are larger while the volumes of V-doped NTO supercells are smaller, than those of NTO supercells, an effect ascribed to the ionic radii of TM ions as discussed previously. To check the cycling stability during charge/discharge processes, we evaluated the relative volume expansion rate *r*_vol_ with respect to the volumes of the host compounds at *x* = 0 as shown in Fig. 2(c). The variation tendencies are similar to those of the cell volumes. Most importantly, the maximal values of *r*_vol_ found at *x* = 1.5 are below 3%, being much smaller than other intercalation-type electrode materials (usually 5 ∼ 6%) as well as alloy-type electrode materials. This indicates that the NTO-related compounds under study can exhibit superior cycling stabilities.

### 3.2 Energetics and electrode potential

**Fig. 3:**
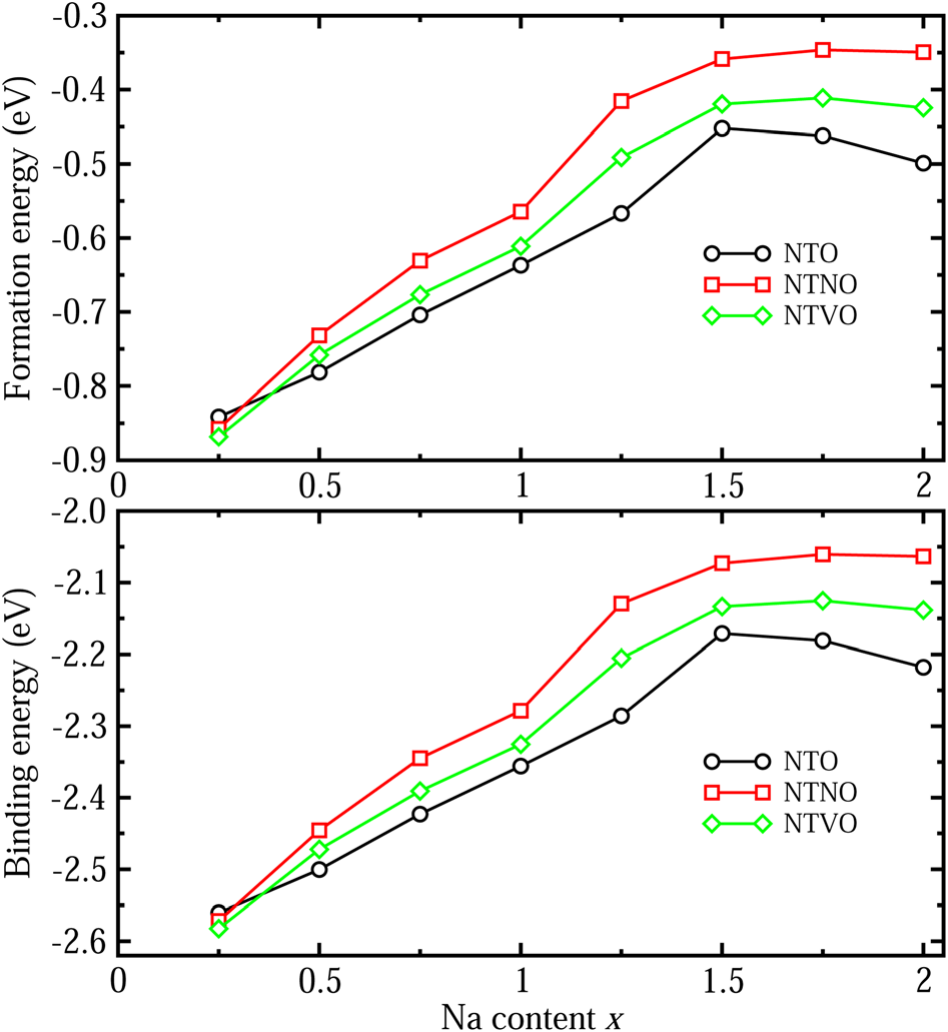
(a) Formation energy per Na atom of the Na-ion insertion intermediate phases resulting in Na_2+*x*_Ti_3_O_7_, Na_2+*x*_Ti,_2.75_Nb,_0.25_O_7_ and Na_2+*x*_Ti,_2.75_V,_0.25_O_7_ with Na content *x* ranging from 0.25 to 2, estimated from the total energies of hosts (*x* = 0) and bcc phase Na bulk, and (b) binding energy per Na atom between the host and inserted Na atom, estimated from the total energies of the hosts and isolated Na atom.

The formability of the intermediate phases can be assessed by calculating their formation energies from the hosts and Na bulk in the body-centered cubic (bcc) phase as follows,^63^1
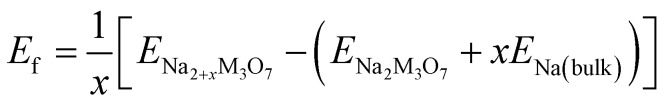
where *E*_Na(bulk)_ is the total energy of the relevant compound and M stands for Ti, Ti,_2.75/3_Nb,_0.25/3_ and Ti,_2.75/3_V,_0.25/3_. The negative value of *E*_f_ indicates spontaneous formation of the intermediate phase from the host and Na bulk, while the positive value is indicative of their endothermic formation, *i.e.*, non-spontaneous formation. Fig. 3(a) displays the calculated formation energy curves as a function of Na content *x*, ranging from 0.25 to 2. It was found that the formation energies varied between −0.9 eV and −0.3 eV, indicating certain formations of the intermediate phases. With increasing Na content *x*, the *E*_f_ values were shown to decrease gradually until *x* = 1.5 and then increase slightly in magnitude, implying the weakening of formability, which is in accordance with the variation of the cell volumes. In the meantime, the formability could be weakened going from NTO to V-doped and to Nb-doped NTO compounds since the *E*_f_ values were decreased in magnitude across the whole Na content range.

To check the binding strength between the host and inserted Na ions, the binding energy *E*_b_ per Na atom was calculated using the following equation,^63^2
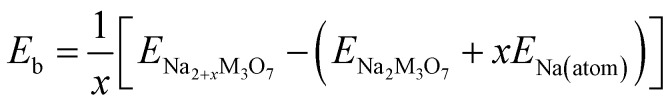
where *E*_Na(atom)_ is the total energy of the isolated Na atom calculated using the cubic supercell with a lattice constant of 15 Å and only the Γ point in the first BZ. As shown in Fig. 3(b), the binding energies could vary between −2.6 eV and −2.0 eV with the same variation tendencies as the formation energies: the binding strength was found to gradually decrease with increasing Na content, with the highest to lowest going from NTO to NTVO and to NTNO.

**Fig. 4:**
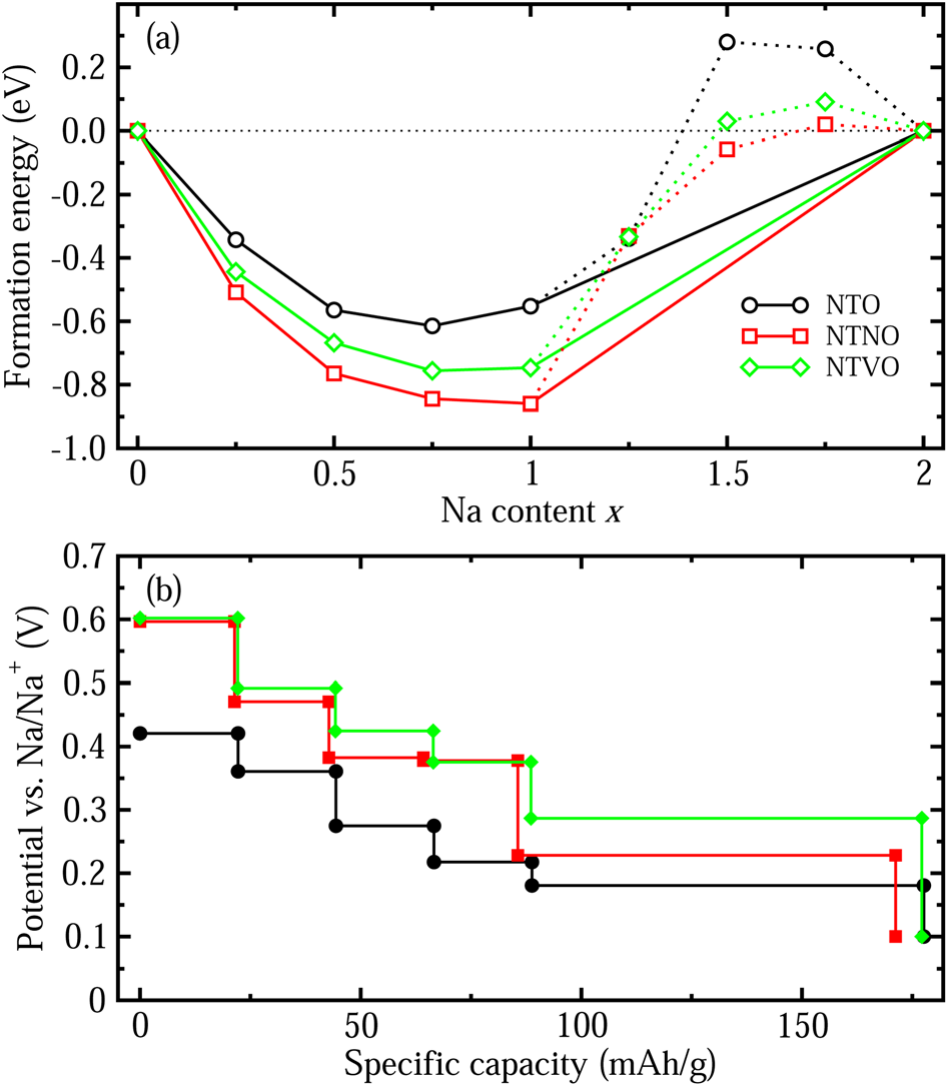
(a) Convex hull plot for formation enthalpies of the intermediate phases from the initial (*x* = 0) and final compounds (*x* = 2) for additional Na insertion in NTO, NTNO and NTVO as a function of Na content *x*. (b) Step-like electrode potential as a function of specific capacity calculated using the formation enthalpies of the stable phases identified by the convex hull.

We also estimated the relative stability of the intermediate phases with respect to the starting (*x* = 0) and ending (*x* = 2) compounds for additional Na insertion. To this end, the formation enthalpies were calculated using the following equation,^64,65^3
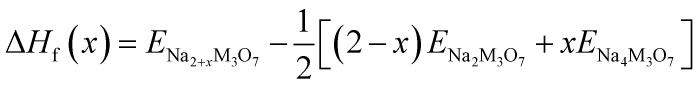
where Na_2_M_3_O_7_ and Na_4_M_3_O_7_ are the initial and final compounds for Na insertion. Fig. 4(a) shows the calculated convex hull curves for the formation enthalpies of NTO, NTNO and NTVO as functions of Na content *x* ranging between 0 and 2. For the three kinds of compounds, the intermediate phases with *x* = 0.25, 0.5, 0.75 and 1.0 were found to be stable because their formation enthalpy values were laid on the convex hull. However, other phases with *x* = 1.25, 1.5 and 1.75 were found to be unstable with respect to the two end compounds, *i.e.*, these phases could not be formed in reality during the charge/discharge process. The intermediate phase with the lowest formation enthalpy was observed at *x* = 0.75 for NTO but at *x* = 1 for the NTNO and NTVO compounds. Moreover, the formation enthalpies of NTNO and NTVO were overall lower compared with NTO due to the weakening of the interactions between cations and anions by Nb or V doping. Also, the Nb-doped NTNO phases exhibited slightly lower formation energies than the V-doped NTVO phases, indicating that the former phases can be formed more easily than the latter ones. This can be associated with the fact that the interaction strength between the Nb^5+,4+^ cations and O^2−^ anions is higher than that between the V^5+,4+^ cations and O^2−^, leading to the enhanced relative stability of the NTNO intermediate phases compared with the NTVO ones.

With these stable intermediate phases identified by the convex hull, we calculated the step-like electrode potential with respect to the Na/Na^+^ counter electrode. The electrode potential between the adjacent phases at *x*_i_ and *x*_j_ could be calculated using the total energies of the relevant phases as follows,^50,64,66^4
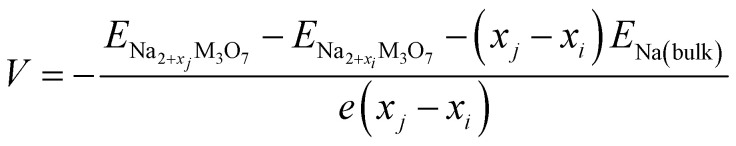
where *e* is the elementary charge. Fig. 4(b) shows the determined step-like electrode potentials for NTO, NTNO and NTVO as functions of specific capacity, which varies between 0 to 177.6, 171.2 and 177.2 mA h g^−1^, respectively. Such reduction of specific capacity with Nb or V doping is due to their heavier atomic weights compared to Ti. It was found that the electrode potential in the plateau region became 0.05 V higher by Nb doping and 0.11 V higher by V doping compared with NTO. The calculated electrode potential steps for NTO and NTNO were in reasonable agreement with the experimentally observed electrode potential profiles.^48^ It is worth noting that the discharge curves look like having long plateau portions, indicating that the materials under study could be used as electrode active materials in SIBs rather than as capacitor electrode materials in SICs. It turned out from our calculations, that the energy density of SIBs employing NTO as the anode material could be slightly lowered by Nb or V doping due to the reduction of specific capacity and the rise in electrode potential.

### 3.3 Electronic properties

**Fig. 5:**
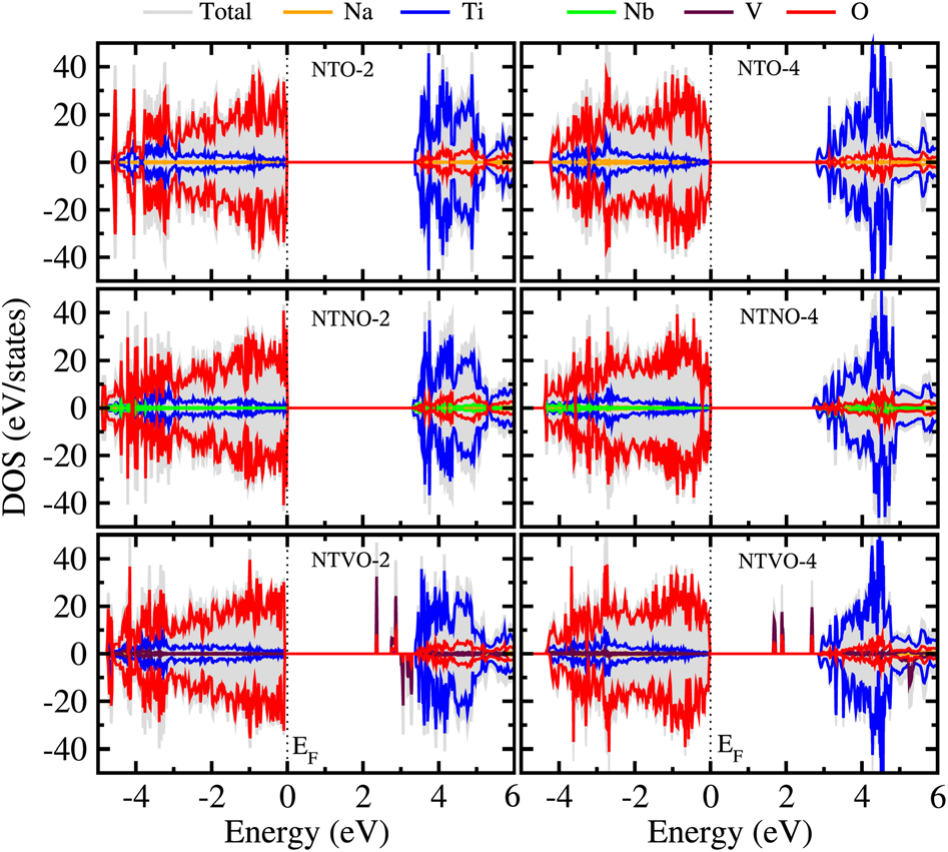
Atomic resolved partial density of states (DOS) in Na_2_Ti_3_O_7_ (NTO-2), Na_4_Ti_3_O_7_ (NTO-4), Na_2_Ti,_2.75_Nb,_0.25_O_7_ (NTNO-2), Na_4_Ti,_2.75_Nb,_0.25_O_7_ (NTNO-4), Na_2_Ti,_2.75_V,_0.25_O_7_ (NTVO-2) and Na_4_Ti,_2.75_V,_0.25_O_7_ (NTVO-4), calculated with the PBEsol functional and DFT + *U* method. Fermi level (*E*_F_) indicated by the vertical dashed line is set to zero.

From previous theoretical and experimental works,^15,33^ it was found that NTO itself has a wide band gap of 3.5–3.7 eV and therefore it is an electrically insulating compound. This is unfavorable for electron transfer during the charge/discharge process, and thus a conductive agent such as carbon black should be added to the electrode. When doping with Nb or V, the band gap can be expected to decrease due to the increased valence electron density, thereby improving the electron conductivity. To check the electronic conduction improvement, we calculated the electronic density of states (DOS) in the compounds. The initial and final compounds Na_2_Ti_3_O_7_ and Na_4_Ti_3_O_7_, denoted as NTO-2 and NTO-4, and their Nb- or V-doped counterparts were selected for electronic structure calculations. Fig. 5 shows the atom-resolved partial DOS of these compounds, calculated using the PBEsol XC functional and DFT + *U* method.

**Fig. 6:**
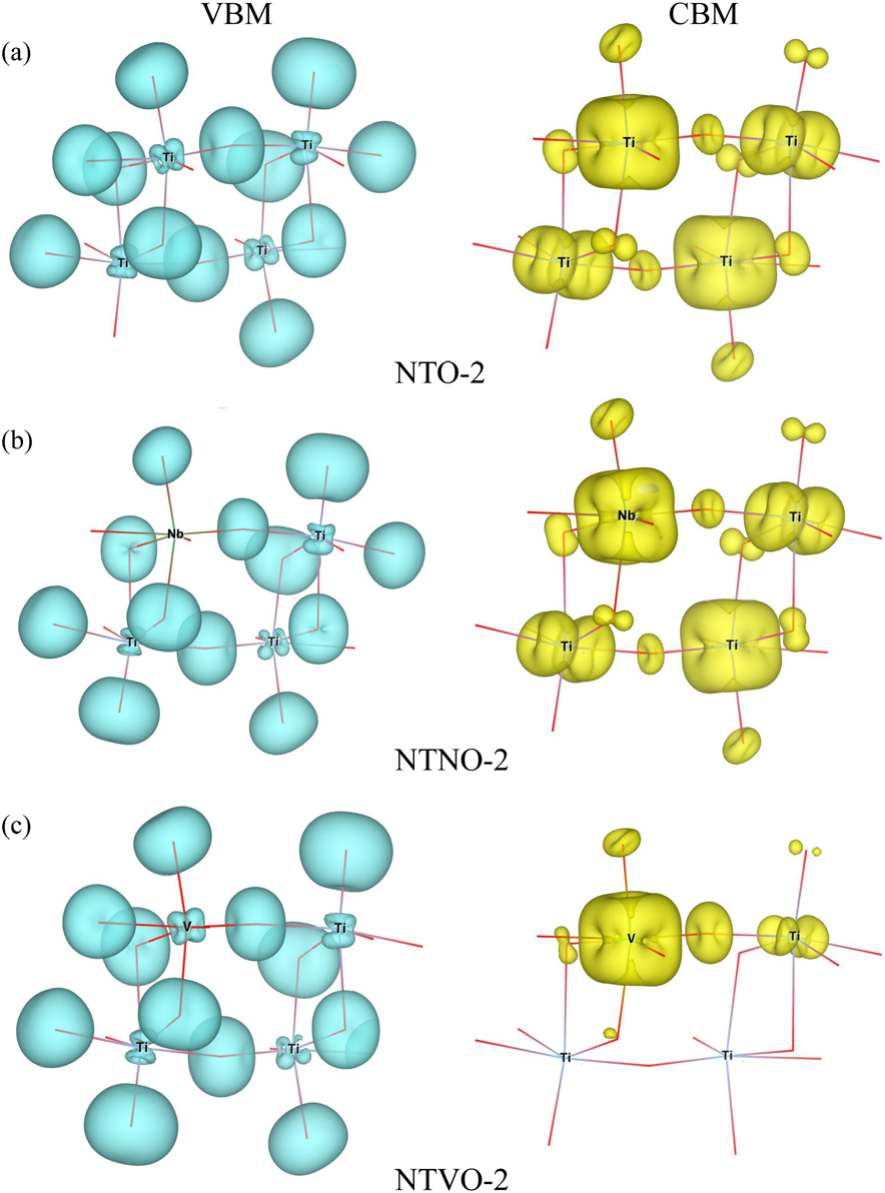
Isosurface plot for integrated local density of states with energies from the valence band maximum (VBM) to VBM −1 eV (left panel), and from conduction band minimum (CBM) to CBM +1 eV (right panel) in (a) Na_2_Ti_3_O_7_ (NTO-2), (b) Na_2_Ti,_2.75_Nb,_0.25_O_7_ (NTNO-2) and (c) Na_2_Ti,_2.75_V,_0.25_O_7_ (NTVO-2).

In our calculations, the band gap of NTO-2 was determined to be 3.37 eV, which is very close to the experimental value. The final compound NTO-4 formed by inserting additional Na ions, showed a lower band gap of 2.78 eV, possibly due to the increased density of conduction band states originating from additional Na ions. When doping with Nb, the band gaps were found to be slightly reduced to lower than 0.1–0.2 eV for both NTNO-2 (3.20 eV) and NTNO-4 (2.68 eV) with almost no preservation of the DOS shape compared with the undoped counterparts. From the orbital-resolved partial DOS analysis, it was found that the conduction band states were dominated by Ti 3d and Nb 4d equally (see Fig. S4 and S5†). Meanwhile, the V-doped compounds, NTVO-2 and NTVO-4, exhibited much lower band gaps of 2.40 and 1.66 eV, respectively, with the characteristic peaks originating from V 3d near the conduction band minimum (CBM) (see also Fig. S6 and S7†). As shown in Fig. 6, for the integrated local density of states, the valence band states close to the valence band maximum (VBM) were composed of O s states with minor contribution from the TM d states, while the conduction band states close to CBM were attributed to the TM d states with minor contribution of the O p states (see Fig. S8†). Based on the calculation results, it can be concluded that the electron conductivity can surely be improved by Nb or V doping.

To get an understanding of electron transfer upon Na ion insertion during the charge/discharge process, we evaluated the Löwdin charges of each atom, as listed in Table S1.† In NTO-2, the average charges were found to be 0.73, 1.5 and −0.8 for Na, Ti and O, respectively. Upon insertion of 8 Na ions, the average charge of Na ion was slightly reduced to 0.7, while oxygen showed a value increased by −0.9. Interestingly, 4 Ti ions kept the charge as ∼1.5 but 8 Ti ions exhibited the reduced value of ∼1.2, confirming the partial reduction of Ti ions upon Na insertion for the discharge process, 3Ti^4+^ → Ti^4+^2Ti^3+^. In the case of NTNO-2, one Ti ion close to Nb had a smaller charge of 1.3 compared to the average value 1.5 in the other Ti ions, implying the reduction of the Ti ion upon Ti/Nb exchange for the sake of charge compensation, *i.e.*, 2Ti^4+^ → Nb^5+^Ti^3+^. When inserting Na ions into NTNO-2, 4 Ti ions also maintained the charge state as 1.5, while 7 Ti ions and Nb ion exhibited a reduction, indicating the partial reduction during the discharge process, 2.5Ti^4+^0.25Ti^3+^0.25Nb^5+^ → Ti^4+^1.75Ti^3+^0.25Nb^4+^. On the contrary, none of the 11 Ti ions had a change in charge state from 1.5 upon Ti/V exchange, thereby indicating that the V ion was in the V^4+^ state. In NTVO-4, 4 Ti ions had an atomic charge of ∼1.5 while the remaining Ti ions and the V ion showed a reduction, thereby indicating the reduction of TM ions like 2.75Ti^4+^0.25V^4+^ → Ti^4+^1.75Ti^3+^0.25V^3+^.

### 3.4 Ion conductivity

**Fig. 7:**
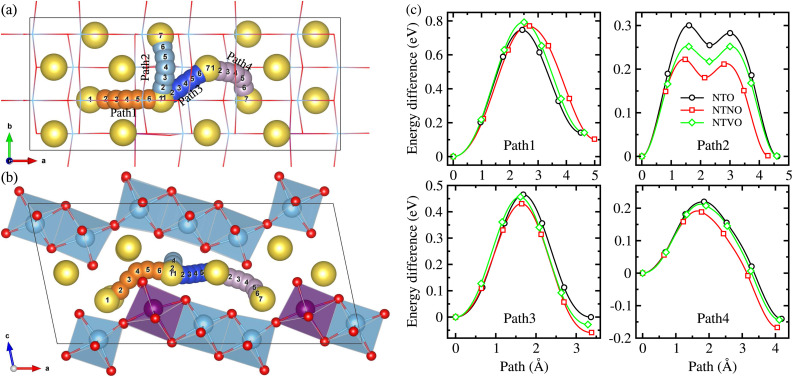
Pathways of Na ion migration within the interlayer space in (a) top and (b) side view, showing 4 different pathways, and (c) calculated energy profiles for Na ion migration along the 4 different pathways according to the vacancy-mediated mechanism in Na_2_M_3_O_7_ (M = Ti, Ti_0.96_Nb_0.04_, Ti_0.96_V_0.04_) compounds.

The Na ion conductivity determines the rate capability and cycling life of SIBs, together with the relative volume expansion rate. Therefore, we investigated the Na ion diffusion, which was expected to occur naturally within the interlayer space and according to the vacancy-mediated mechanism like in most insertion-type layered electrode materials. The 2 × 2 × 1 supercells were used to get more reasonable insight into the Na ion conduction. As shown in Fig. 7(a) and (b), we suggested 4 different pathways to consider all the possible ways for the Na ion migration within the interlayer space; (1) pathway of ascending the step in the (100) direction (Path 1), pathway of horizontal moving on the step in the (010) direction (Path 2), pathway of diagonal moving on the step in the (110) direction (Path 3 and Path 4). We estimated the activation energies for Na ion diffusion along these pathways by applying the NEB method. It should be noted that the Na ion diffusion across the layer was not allowed, undoubtedly due to obstacles formed by the MO_6_ octahedra and thus much higher activation energy.^33^

Fig. 7(c) displays the calculated energy profiles for Na ion migration along these suggested pathways. Along Path 1, the activation energies were determined to be 0.75, 0.77 and 0.79 eV for NTO, NTNO and NTVO, respectively. These are relatively high compared with those along other pathways, indicating that the Na migration ascending the step could not occur in reality. The horizontal Na ion migration along the (010) direction (Path 2) produced the energy difference curves with a two-peaks feature, for which the activation energies were calculated to be 0.30, 0.25 and 0.22 eV for NTO, NTVO and NTNO, respectively. For the case of NTO, although slightly underestimated, the calculated value was in good agreement with the experimental value of 0.39 eV.^67^ The reduction of activation energy by Nb or V doping, indicating the improvement of Na ion conductivity, could be attributed to the enhancement of cation–cation repulsion. Meanwhile, along Path 3 in the diagonal direction, the activation energy was found to be 0.47 eV for NTO and slightly reduced by Nb or V doping. Also, the activation energy along Path 4 was reduced from 0.22 eV for NTO to 0.21 eV by V doping and to 0.19 eV by Nb doping. To sum up, the Na ion conductivity on the step layer can be enhanced by V doping and furthermore by Nb doping. Such enhancement of Na ion conductivity by Nb or V doping can be ascribed to the enhancement of repulsion between the Na cation and TM cations, occurring the charge state change of 2Ti^4+^ → Nb^5+^Ti^3+^ or V^4+^Ti^4+^.

Finally we estimated the diffusion coefficients using the calculated activation energies *E*_a_ and the Arrhenius equation, *D* = *a*^2^*ν* exp(−*E*_a_/*k*_B_*T*) with *a* ≈ 4 Å, *ν* ≈ 10^11^ Hz and *k*_B_*T* = 0.026 eV at room temperature.^33^ The activation energies for Na ion migration only, along Path 2 were considered here. Table 2 presents the calculated diffusion coefficients of NTO, NTNO and NTVO, together with other electrochemical properties determined in this work. They exhibited relatively high diffusion coefficients of 1.54 × 10^−9^, 3.09 × 10^−8^ and 9.91 × 10^−9^ cm^2^ s^−1^ along the horizontal path, confirming the enhancement of Na ion diffusibility by Nb or V doping. As presented in Table 2, the relative volume expansion rate, specific capacity and electrode potential, could be weakened but the electronic and ionic conductivities could be enhanced by Nb or V doping.

**Table 2:** Material properties of Na_2_Ti_3_O_7_ (NTO), Nb-doped NTO (NTNO) and V-doped NTO (NTVO), calculated in this work

Properties	NTO	NTNO	NTVO
Volume expansion rate (%)	2.29	2.72	2.36
Specific capacity (mA h g^−1^)	177.6	171.2	177.2
Electrode potential (V)	0.18	0.23	0.29
Band gap (eV)	3.37	3.20	2.40
Activation energy (eV)	0.30	0.22	0.25
Diffusion coefficient (× 10^−9^ cm^2^ s^−1^)	1.54	30.87	9.91

## 4 Conclusions

In this work, we have investigated the effect of Nb or V doping into sodium titanate Na_2_Ti_3_O_7_, on the electrode performance using first-principles calculations, aiming to find advanced anode materials for sodium-ion batteries. We made materials models for Nb- or V-doped NTO using the doubled supercells, forming Na_2_Ti,_2.75_M,_0.25_O_7_ (M = Nb, V), to investigate the structural, electrochemical and electronic properties, and the 2 × 2 × 1 supercells for studying the Na ion diffusion. From structural optimization, it was found that the Nb doping increased the supercell volume with the relative volume expansion rate *r*_vol_ of 0.9% due to the larger ionic radius of the Nb ion, whereas the V doping shrank the cell volume with the *r*_vol_ value of −0.4% because of the smaller ionic radius of the V ion. Through ΔBVS analysis, the final sodiation compound was fixed as Na_4_M_3_O_7_ and the positions for inserting Na ions into the interlayer space were identified. We performed structural optimizations of the intermediate phases Na_2+*x*_M_3_O_7_, with increasing Na content *x* from 0 to 2, finding that the intermediate phases could be formed exothermically from the NTO and Na bulk as the negative formation energies and maximum *r*_vol_ values could be slightly increased by Nb or V doping, but remained lower than 3%. The formation enthalpies of the intermediate phases were calculated with respect to the initial (NTO-2) and final (NTO-4) compounds, to plot the convex hull and electrode potential curves, revealing that the electrode potential could be slightly raised and the specific capacity could be reduced by Nb or V doping. Through DOS analysis, it was found that the band gap of 3.37 eV in NTO was reduced to 3.20 eV in NTNO-2 and to 2.40 eV in NTVO-2, which was ascribed to the 4d state of Nb and 3d state of V, indicating the improvement of electronic conductivity by doping. The redox couples during sodiation were identified through the analysis of the Löwdin charge of the atoms. Finally, we calculated the activation energies for the vacancy-mediated Na ion migration and found significant improvement of Na ion conductivity from Nb or V doping.

## Author contributions

Suk-Gyong Hwang performed the calculations and drafted the first manuscript. Chung-Hyok Kim performed the post-processing of calculation results. Song-Hyok Choe and Kum-Chol Ri assisted with the DFT calculations and contributed to useful discussions. Chol-Jun Yu developed the original project and supervised the work. All authors reviewed the manuscript.

## Conflicts of interest

There are no conflicts to declare.

## Supplementary Material

RA-013-D3RA01755A-s001
