# Supplementary material for: Revealing the effect of Nb or V doping on anode performance in Na2Ti3O7 for sodium-ion batteries: a first-principles study
Source: RSC Adv. 2023 Jun 5;13(24):16749–57. doi: 10.1039/d3ra01755a (PMC10240172; doi:10.1039/d3ra01755a)
Supplement: RA-013-D3RA01755A-s001 [file RA-013-D3RA01755A-s001.pdf]

Electronic Supplementary Material (ESI) for RSC Advances.
This journal is © The Royal Society of Chemistry 2023

Supporting Information – Revealing the effect of Nb or V doping on anode performance in $Na_2Ti_3O_7$ for sodium-ion battery: A first-principles study

Suk-Gyong Hwang, Chung-Hyok Kim, Song-Hyok Choe, Kum-Chol Ri, Chol-Jun Yu*

Computational Materials Design (CMD), Faculty of Materials Science, Kim Il Sung University, Ryongnam-Dong, Taesong District, Pyongyang, PO Box 76, Democratic People's Republic of Korea

*Corresponding author: Chol-Jun Yu, Email: cj.yu@ryongnamsan.edu.kp

Table S1. Löwdin charge of atoms in $Na_2TiO_3O_7$ (NTO-2), $Na_4Ti_3O_7$ (NTO-4), $Na_2Ti_{2.75}Nb_{0.25}O_7$ (NTNO-2), $Na_4Ti_{2.75}Nb_{0.25}O_7$ (NTNO-4), $Na_2Ti_{2.75}V_{0.25}O_7$ (NTVO-2) and $Na_4Ti_{2.75}V_{0.25}O_7$ (NTVO-4).

Atoms	NTO-2	NTO-4	NTNO-2	NTNO-4	NTVO-2	NTVO-4
Na	0.7314	0.7015	0.7376	0.7415	0.7318	0.7004
Na	0.7319	0.7124	0.7500	0.7452	0.7309	0.7127
Na	0.7314	0.7016	0.7744	0.7453	0.7383	0.7043
Na	0.7319	0.7124	0.7333	0.7136	0.7321	0.7128
Na	0.7314	0.7016	0.7311	0.7019	0.7319	0.7025
Na	0.7319	0.7124	0.7350	0.7137	0.7310	0.7130
Na	0.7314	0.7016	0.7744	0.7453	0.7383	0.7042
Na	0.7319	0.7124	0.7333	0.7136	0.7321	0.7128
Ti	1.5185	1.2713	1.5156	1.3264	1.5137	1.3855
Ti	1.4958	1.4807	1.4698	1.5326	1.4982	1.5927
Ti	1.5184	1.2714	1.5508	1.3542	1.5197	1.3374
Ti	1.4958	1.4804	1.5292	1.5069	1.4966	1.5358
Ti	1.5674	1.2050	1.5446	1.2953	1.5673	1.2930
Ti	1.5184	1.2706	1.4853	1.3281	1.5134	1.3275
Ti	1.4958	1.4801	1.4555	1.5372	1.4978	1.4996
Ti	1.5674	1.2035	1.3648	1.0839	1.5500	1.3643
Ti	1.5185	1.2704	1.5508	1.3441	1.5197	1.3369
Ti	1.4958	1.4804	1.5292	1.5021	1.4966	1.5352
Ti	1.5674	1.2036	1.5446	1.2821	1.5673	1.2926
Ti, Nb, V	1.5674	1.2053	0.1288	0.0101	1.0556	0.2759
O	−0.7750	−0.8936	−0.5616	−0.7551	−0.7504	−0.9228
O	−0.7757	−0.8908	−0.4980	−0.6878	−0.5784	−0.9924
O	−0.7976	−0.8437	−0.7636	−0.8362	−0.7633	−0.8457
O	−0.8195	−0.8885	−0.7827	−0.8769	−0.8085	−0.8819
O	−0.8362	−0.9175	−0.8213	−0.9104	−0.8284	−0.9168
O	−0.8387	−0.9037	−0.6982	−0.7684	−0.7892	−0.9137
O	−0.8329	−0.9089	−0.7272	−0.7940	−0.8010	−0.8974
O	−0.7750	−0.8936	−0.7595	−0.8914	−0.7705	−0.8898
O	−0.7757	−0.8908	−0.7523	−0.8852	−0.7660	−0.8892
O	−0.7976	−0.8437	−0.7529	−0.8463	−0.7945	−0.8351
O	−0.8194	−0.8885	−0.8062	−0.8854	−0.8158	−0.8846
O	−0.8362	−0.9175	−0.6529	−0.7955	−0.8103	−0.9057
O	−0.8387	−0.9037	−0.8246	−0.9005	−0.8271	−0.9002
O	−0.8329	−0.9089	−0.8169	−0.9065	−0.8290	−0.9083
O	−0.7750	−0.8936	−0.7425	−0.8984	−0.7744	−0.8917
O	−0.7757	−0.8908	−0.7583	−0.8912	−0.7674	−0.8915
O	−0.7976	−0.8437	−0.7935	−0.8392	−0.7938	−0.8399
O	−0.8194	−0.8886	−0.8157	−0.8856	−0.8154	−0.8869
O	−0.8362	−0.9175	−0.8284	−0.9154	−0.8313	−0.9114
O	−0.8387	−0.9037	−0.8241	−0.9093	−0.8378	−0.8918
O	−0.8329	−0.9089	−0.8210	−0.9051	−0.8294	−0.9029
O	−0.7750	−0.8936	−0.7595	−0.8912	−0.7705	−0.8898
O	−0.7757	−0.8908	−0.7523	−0.8851	−0.7660	−0.8897
O	−0.7976	−0.8437	−0.7529	−0.8460	−0.7945	−0.8347
O	−0.8195	−0.8886	−0.8062	−0.8854	−0.8158	−0.8845
O	−0.8362	−0.9175	−0.6529	−0.7958	−0.8103	−0.9058
O	−0.8387	−0.9037	−0.8246	−0.9006	−0.8271	−0.9004
O	−0.8329	−0.9089	−0.8169	−0.9067	−0.8290	−0.9083
Na		0.7111		0.7423		0.7131
Na		0.7111		0.7423		0.7131
Na		0.7249		0.7620		0.7265
Na		0.7249		0.7262		0.7258
Na		0.7249		0.7261		0.7254
Na		0.7249		0.7261		0.7254
Na		0.7111		0.7167		0.7110
Na		0.7111		0.7120		0.7114

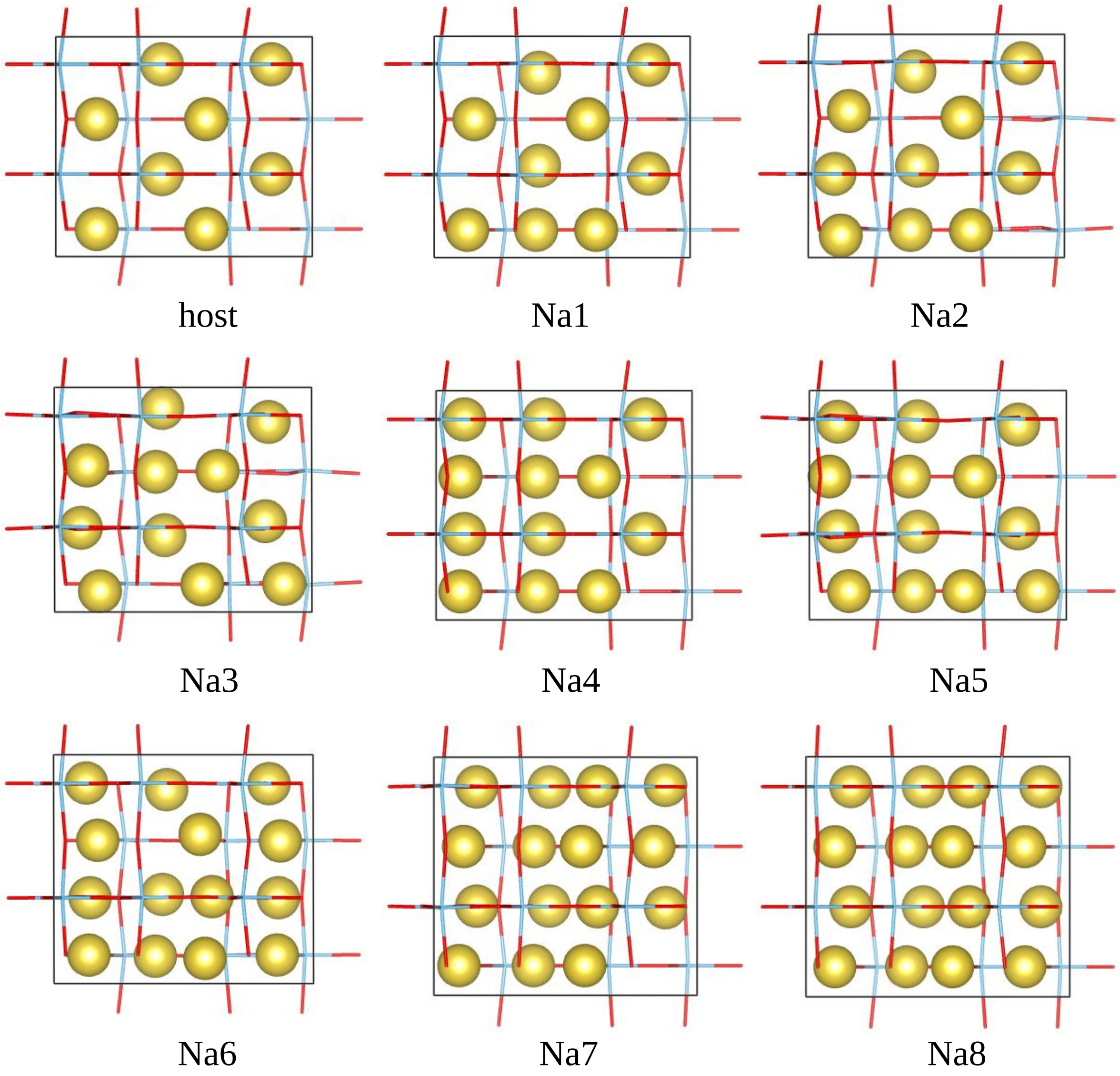

Figure S1. Top view of optimized structures for the lowest energy configuration for $Na_{2+x}Ti_3O_7$ (x = 0, 0.25, 0.5, 0.75, 1, 1.25, 1.5, 1.75, 2).

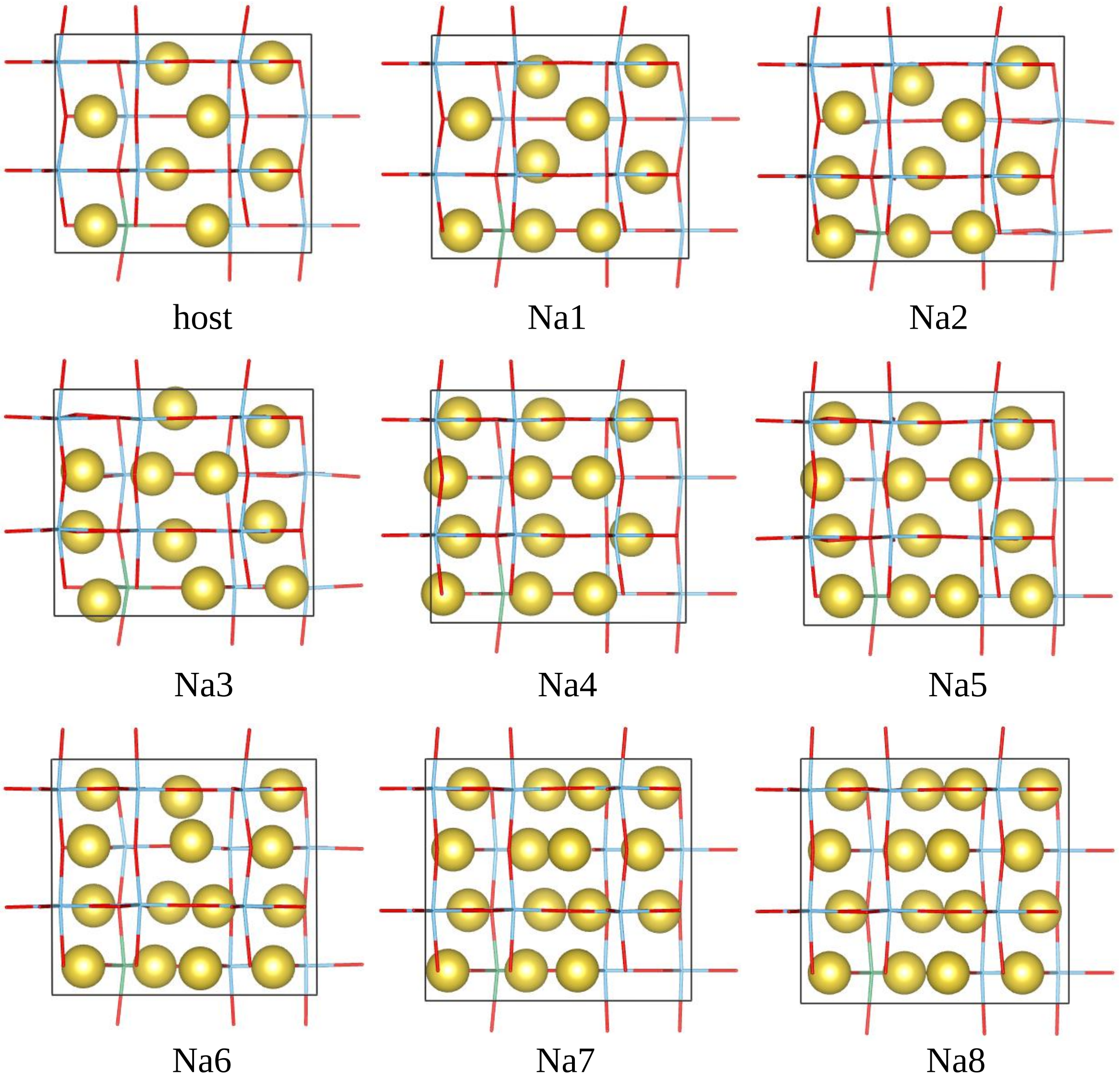

Figure S2. Top view of optimized structures for the lowest energy configuration for $Na_{2+x}Ti_{2.75}Nb_{0.25}O_7$ (x = 0, 0.25, 0.5, 0.75, 1, 1.25, 1.5, 1.75, 2).

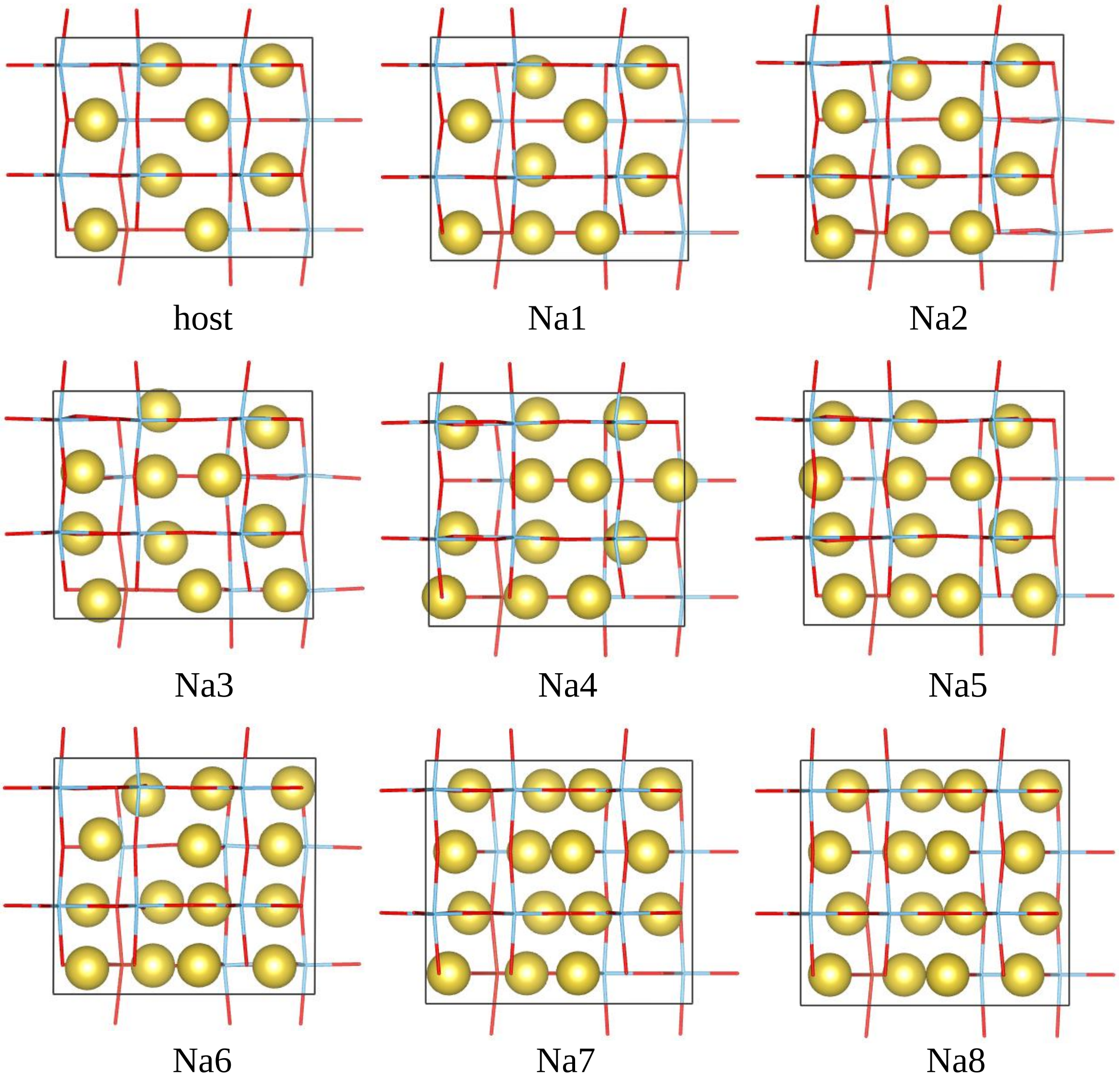

Figure S3. Top view of optimized structures for the lowest energy configuration for $Na_{2+x}Ti_{2.75}V_{0.25}O_7$ (x = 0, 0.25, 0.5, 0.75, 1, 1.25, 1.5, 1.75, 2).

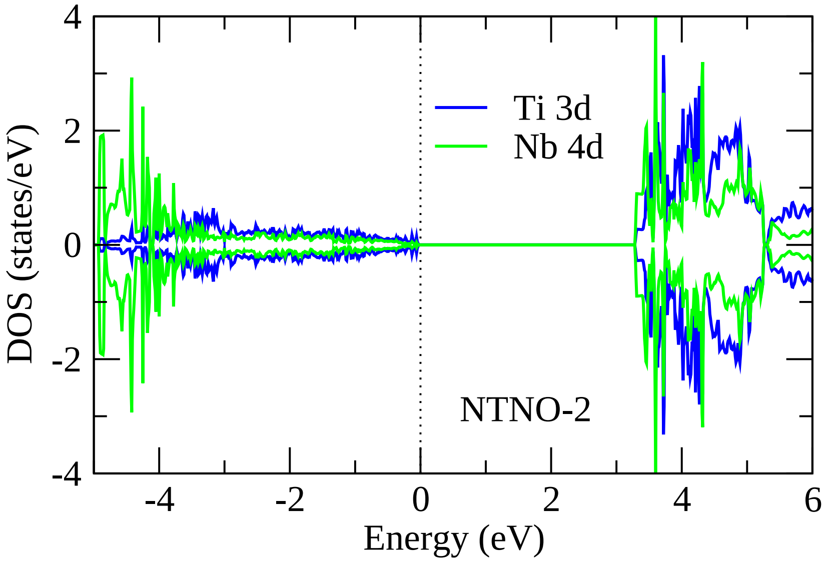

Figure S4. Orbital-resolved partial density of states for Ti 3d and Nb 4d in $Na_2Ti_{2.75}Nb_{0.25}O_7$.

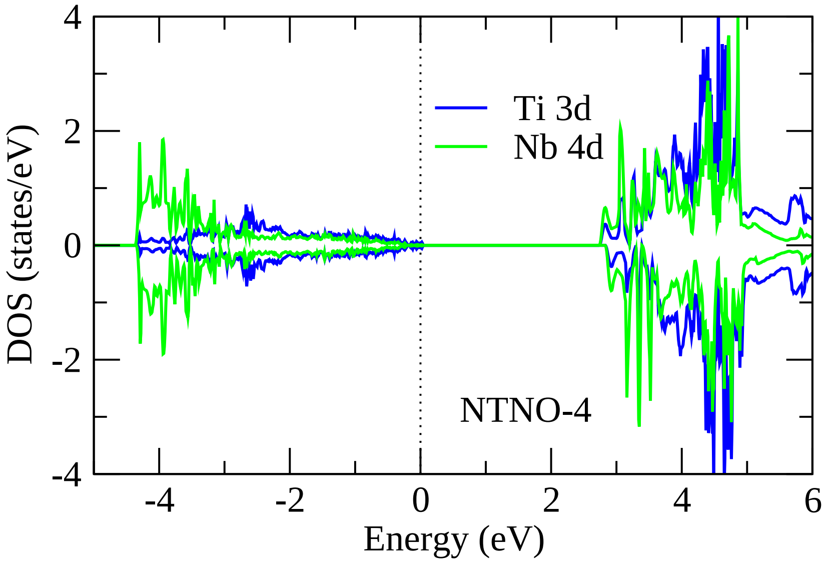

Figure S5. Orbital-resolved partial density of states for Ti 3d and Nb 4d in $Na_4Ti_{2.75}Nb_{0.25}O_7$.

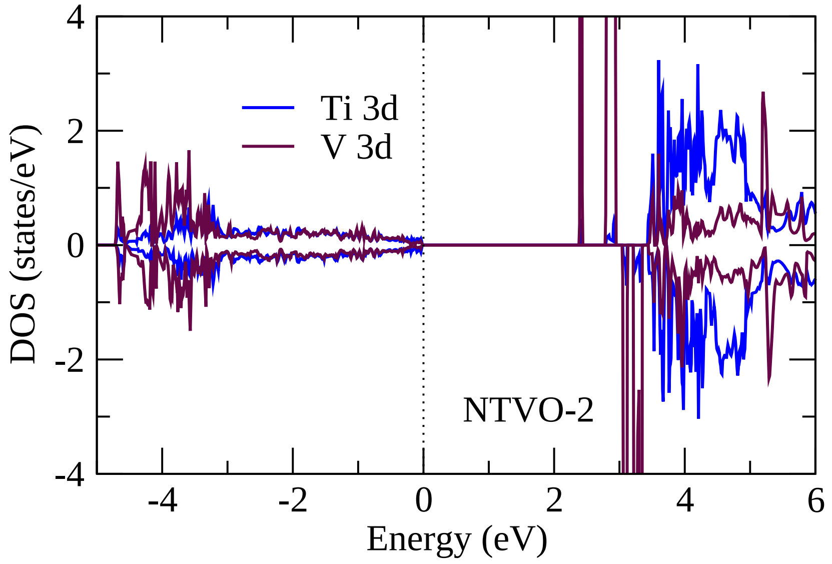

Figure S6. Orbital-resolved partial density of states for Ti 3d and V 3d in $Na_2Ti_{2.75}V_{0.25}O_7$.

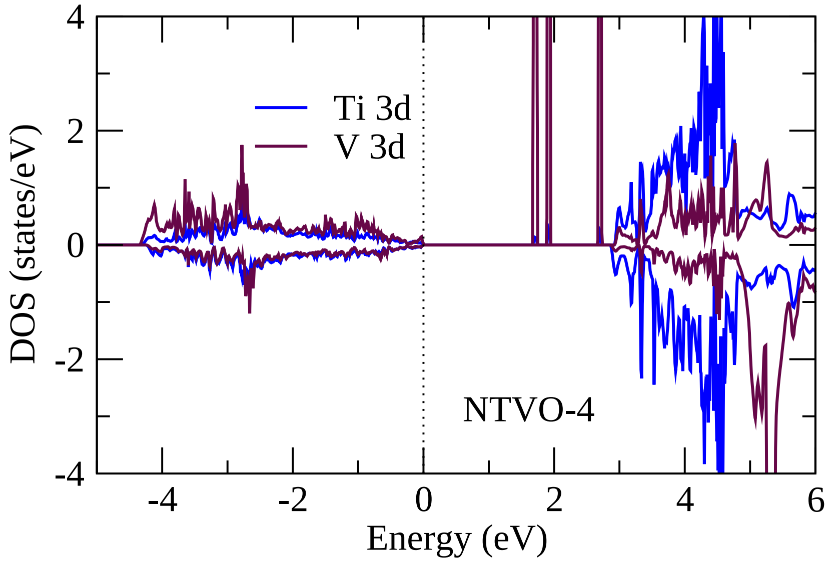

Figure S7. Orbital-resolved partial density of states for Ti 3d and V 3d in $Na_4Ti_{2.75}V_{0.25}O_7$.

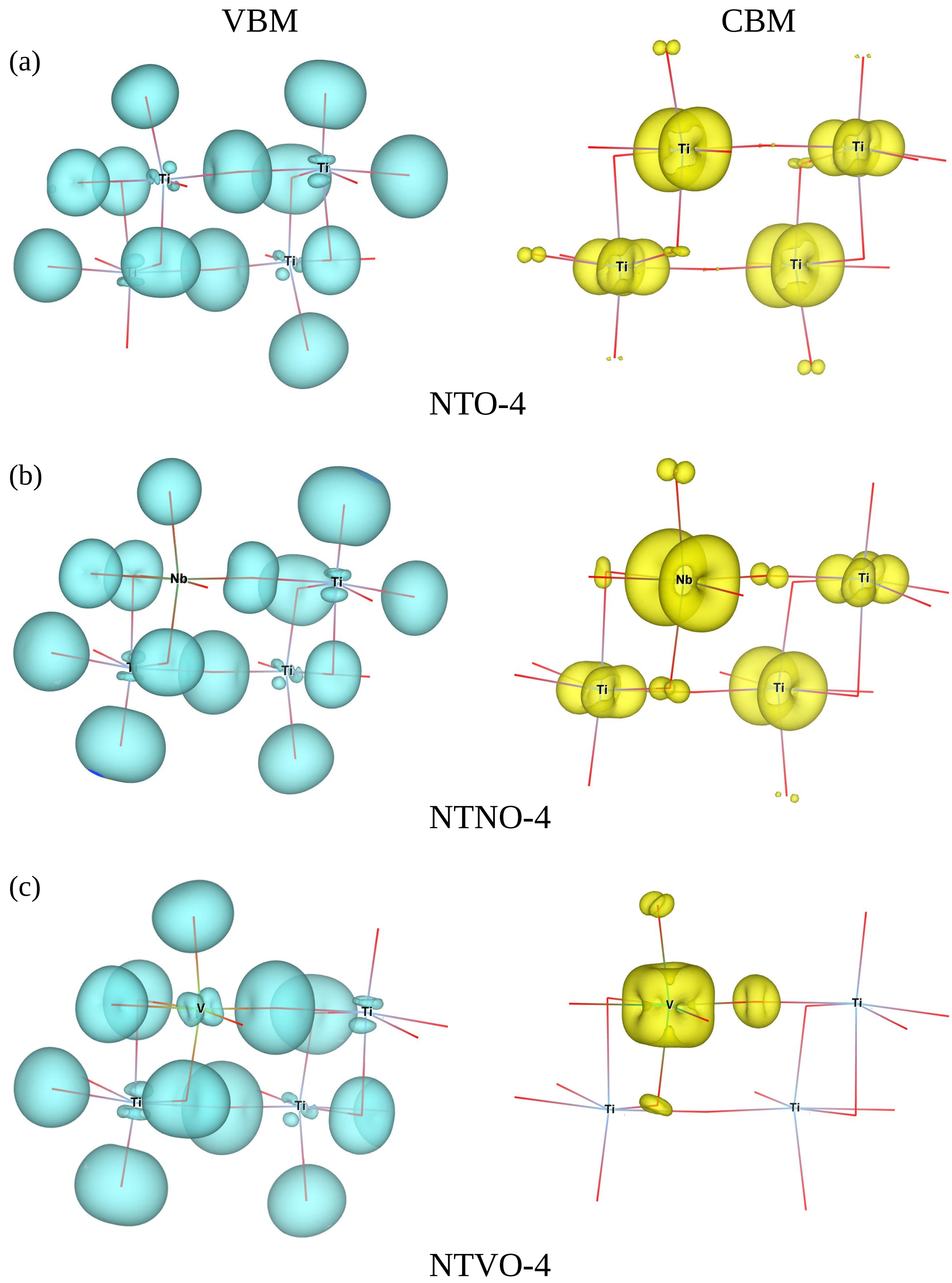

Figure S8. Isosurface plot for integrated local density of states with energies from valence band maximum (VBM) to VBM-1 eV (left panel), and from conduction band minimum (CBM) to CBM+1 eV (right panel) in $Na_4Ti_3O_7$ (NTO-4), $Na_4Ti_{2.75}Nb_{0.25}O_7$ (NTNO-4) and $Na_4Ti_{2.75}V_{0.25}O_7$ (NTVO-4).
